# Caloric restriction increases brain mitochondrial calcium retention capacity and protects against excitotoxicity

**DOI:** 10.1111/acel.12527

**Published:** 2016-09-13

**Authors:** Ignacio Amigo, Sergio Luiz Menezes‐Filho, Luis Alberto Luévano‐Martínez, Bruno Chausse, Alicia J. Kowaltowski

**Affiliations:** ^1^ Departamento de BioquímicaInstituto de QuímicaUniversidade de São PauloSão PauloBrazil

**Keywords:** aging, brain, calcium, caloric restriction, mitochondria

## Abstract

Caloric restriction (CR) protects against many cerebral pathological conditions that are associated with excitotoxic damage and calcium overload, although the mechanisms are still poorly understood. Here we show that CR strongly protects against excitotoxic insults *in vitro* and *in vivo* in a manner associated with significant changes in mitochondrial function. CR increases electron transport chain activity, enhances antioxidant defenses, and favors mitochondrial calcium retention capacity in the brain. These changes are accompanied by a decrease in cyclophilin D activity and acetylation and an increase in Sirt3 expression. This suggests that Sirt3‐mediated deacetylation and inhibition of cyclophilin D in CR promote the inhibition of mitochondrial permeability transition, resulting in enhanced mitochondrial calcium retention. Altogether, our results indicate that enhanced mitochondrial calcium retention capacity underlies the beneficial effects of CR against excitotoxic conditions. This protection may explain the many beneficial effects of CR in the aging brain.

## Introduction

Mechanisms that increase longevity and, perhaps most importantly, promote longer health spans (lower or delayed incidence of age‐related diseases) have always attracted attention. The most effective intervention known to date to prevent age‐related decline and promote better health spans in a wide variety of organisms, ranging from yeast to primates, is caloric restriction (CR). This dietary intervention typically consists of a 20–40% reduction in caloric intake without micronutrient limitation relative to an *ad libitum* diet. CR benefits in vertebrates include the prevention of metabolic, cardiac, vascular, proliferative, and neurological diseases (Fontana & Partridge, 2015).

Perhaps the most striking group of age‐related diseases prevented by CR is in the brain. A large number of neurological disorders are age‐related, and CR has been demonstrated to effectively prevent these disorders (Amigo & Kowaltowski, 2014). CR also improves age‐related declines in memory and learning abilities observed in elderly animals (Stewart *et al*., 1989; Witte *et al*., 2009). Although the mechanisms by which CR exerts its effects are poorly understood, mitochondria, as master regulators of cellular metabolism, are believed to play an important role in the cellular adaptations that take place with the diet. While some initial work suggested that CR increases mitochondrial mass, conflicting results have cast doubts over the validity of this observation (Hancock *et al*., 2011; Lanza *et al*., 2012). However, mitochondrial function may be upregulated independently of increases in mass: CR has been shown to promote deacetylation of a large number of mitochondrial proteins in a tissue‐specific manner, mainly through the deacetylase Sirt3, suggesting it can act as a molecular trigger to promote oxidative phosphorylation under fasting or CR conditions (Lombard *et al*., 2007).

In the brain, increases in mitochondrial activity may change the susceptibility to excitotoxicity, a pathological process associated with many age‐related neurological conditions such as stroke, Alzheimer's disease and Parkinson's disease, in which excessive activation of postsynaptic receptors results in cell death (Nicholls, 2009). This neurodegenerative process involves the binding of glutamate or glutamate analogues to NMDA and AMPA receptors, resulting in pathological increases in cytosolic calcium levels and a rapid decrease in ATP levels due to the activation of ionic balance restoration pathways. Mitochondria are the main site for ATP production in neurons and contribute toward cellular calcium buffering by accumulating this ion in a membrane potential‐dependent manner. Indeed, interventions that increase mitochondrial calcium buffering capacity protect against excitotoxicity and related conditions (Schinzel *et al*., 2005; Li *et al*., 2009).

Interestingly, while intermittent fasting (a dietary intervention that consists in offering food *ad libitum* on alternate days) has been found to be neuroprotective under excitotoxic conditions (Bruce‐Keller *et al*., 1999; Contestabile *et al*., 2004; Sharma & Kaur, 2005), the effects of CR on excitotoxicity have not been well explored to date. Furthermore, mechanistic insights toward possible neuroprotective effects of this diet are still scarce. The aim of this study was to determine the effects of CR on excitotoxicity and dissect the molecular mechanisms involved.

## Results

### CR protects against excitotoxicity *in vivo*

**Figure 1:**
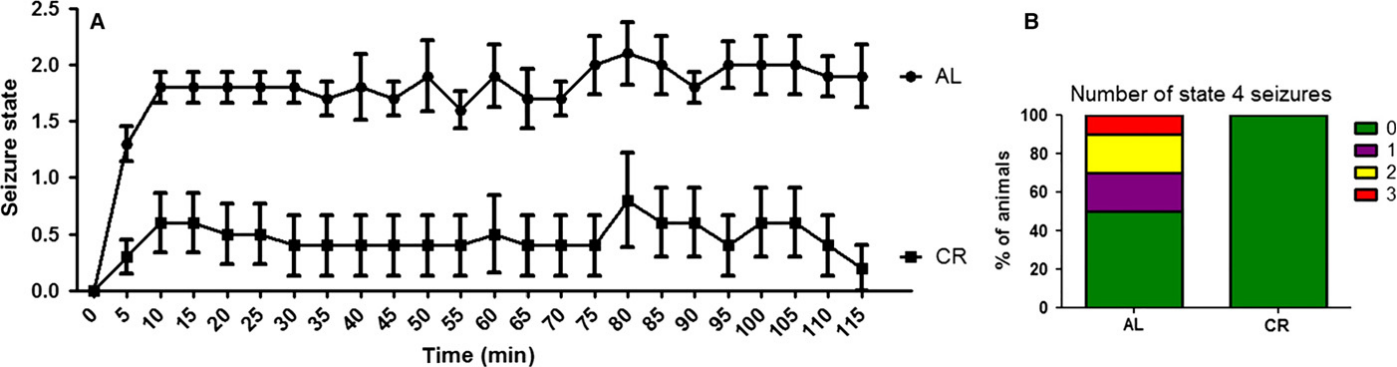
Caloric restriction protects against *in vivo* excitotoxicity. (A) Time course of seizure appearance in AL and CR animals after 25 mg kg^−1^ kainic acid i.p. injection. (B) Number of state 4 seizures measured using Racine's scale. Results are shown as mean ± SEM.

Intermittent fasting has been shown to protect against excitotoxic damage (Contestabile *et al*., 2004; Sharma & Kaur, 2005), but the effects of CR on excitotoxicity have not been evaluated. Because CR and intermittent fasting can present very distinct hypothalamic effects (Froy & Miskin, 2010), we sought to evaluate whether CR also affected this form of neuronal damage. Male Swiss mice maintained for 14 weeks on a CR diet were injected with kainic acid, a glutamate analogue which causes brain damage, seizures, and neuronal cell death due to overactivation of glutamate receptors in the hippocampus. We found that CR animals displayed a striking resistance to kainic acid‐induced seizures, presenting far less seizures over time (Fig. 1A) and completely avoiding the occurrence of more intense state 4 seizures (Fig. 1B). These results demonstrate that the CR diet can strongly protect against excitotoxicity.

### CR brains present enhanced antioxidant activity

**Figure 2:**
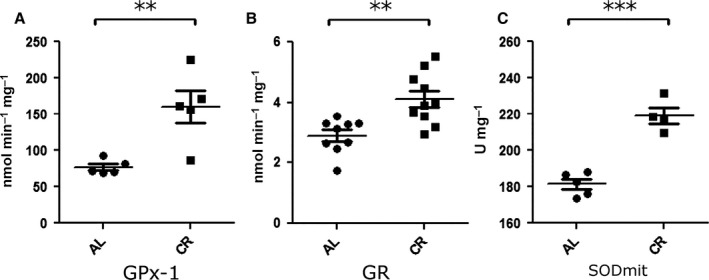
Antioxidant activity is higher in brains from CR animals. The activity of antioxidant enzymes was assayed in whole‐brain homogenates (A, B) or mitochondrial fractions (C). (A) Enzymatic activity of glutathione peroxidase (Gpx‐1). (B) Enzymatic activity of glutathione reductase (GR). (C) Enzymatic activity of superoxide dismutase in mitochondrial fractions (SOD mit). Results are shown as mean ± SEM. **P < 0.01; ***P < 0.001 two‐tailed, unpaired Student's *t*‐test.

Excitotoxic cell death involves oxidative imbalance (Nicholls, 2009). To explore how CR affects brain redox state, we used Sprague Dawley rats, which allowed us to obtain larger tissue samples. We observed an increase in the activity of antioxidant enzymes such as glutathione peroxidase 1 (Fig. 2A) and glutathione reductase (Fig. 2B) in whole‐brain homogenates from animals fed a CR diet relative to AL. SOD activity in mitochondrial fractions was also higher in CR samples (Fig. 2C). Together, these results indicate that the redox capacity of CR brains is largely enhanced, preparing these organs for oxidative damage conditions such as excitotoxicity.

### Caloric restriction enhances mitochondrial electron transport

**Figure 3:**
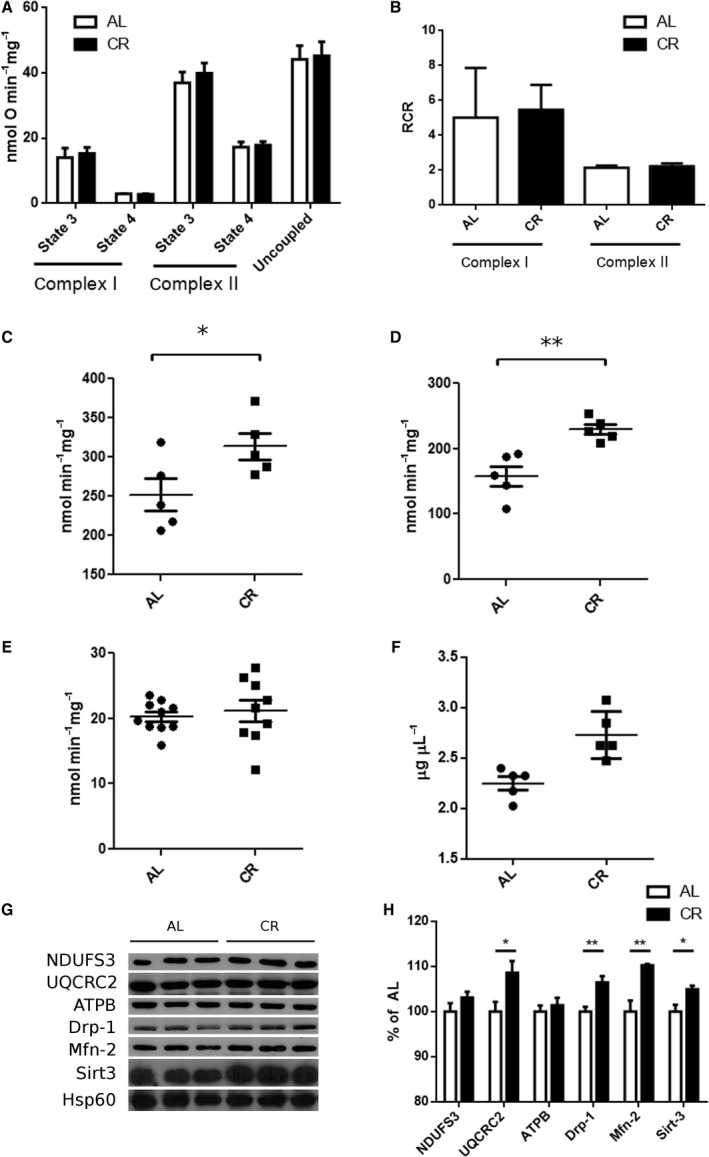
CR induces mitochondrial changes in the brain. (A) Oxygen consumption in isolated brain mitochondria. Rates are shown in the presence (state 3) and absence (state 4) of ADP, both with substrates of complex I and complex II. Uncoupled rates were determined after addition of CCCP. (B) Respiratory control rates determined as the ratio state 3/state 4. (C) Enzymatic activity of complex I + III in mitochondrial samples. (D) Enzymatic activity of complex IV in mitochondrial samples. (E) Citrate synthase activity in whole‐brain homogenates. (F) Cardiolipin determination in whole‐brain homogenates. (G) Western blot of mitochondrial samples using antibodies against different mitochondrial proteins: NDUFS3 (complex I), UQCRC2 (complex III), ATPB (complex V), Drp‐1, Mfn‐2, Sirt3, and Hsp60. (H) Quantification of bands from G using Hsp60 as loading control. Results of CR samples are shown as percentage of AL samples. Results are shown as mean ± SEM. *P < 0.05 two‐tailed, **P < 0.01, two‐tailed unpaired Student's t‐test.

Mitochondria play a key role in both excitotoxic cell damage and redox maintenance (Nicholls, 2009). Furthermore, CR has been widely shown to alter mitochondrial bioenergetics in a wide variety of tissues, including the brain (Lin *et al*., 2014). Based on this and the enhanced SOD activity observed in mitochondria (Fig. 2C), we investigated whether CR promoted changes in mitochondria which could account for the protection against excitotoxicity observed. We did not detect changes in isolated mitochondrial respiration using either complex I or complex II substrates (Fig. 3A,B). However, we found that CR brains present higher activities of complexes I+III (Fig. 3C) and complex IV (Fig. 3D). We also assessed mitochondrial mass in the cerebral tissue using two methods: While we did not detect any changes in the activity of the matrix enzyme citrate synthase (Fig. 3E) with CR, we observed an increase in the concentration of cardiolipin, a lipid found specifically in the inner mitochondrial membrane (Fig. 3F). Next, we measured the levels of a panel of mitochondrial proteins (Fig. 3G): NDUFS3 (a complex I component) and UQCRC2 (complex III), ATPB (β‐units of the ATP synthase), Drp‐1 (a protein involved in mitochondrial fission), Mfn‐2 (a protein involved in mitochondrial fusion), and Sirt3 (a mitochondrial deacetylase from the sirtuin family) were normalized against Hsp60, a mitochondrial protein with no described bioenergetic role (Fig. 3H). We found a significant increase in UQCRC2, Drp‐1, and Mfn‐2. Similarly to what has been described for other tissues (Shi *et al*., 2005; Palacios *et al*., 2009), CR increased the levels of Sirt3, whereas ATPB and NDUFS3 did not show significant changes. These results indicate that CR affects brain mitochondria by increasing the levels of specific proteins and mitochondrial electron transport chain activity.

### Serum from CR animals stimulates mitochondrial activity and protects against glutamate‐induced cell death

**Figure 4:**
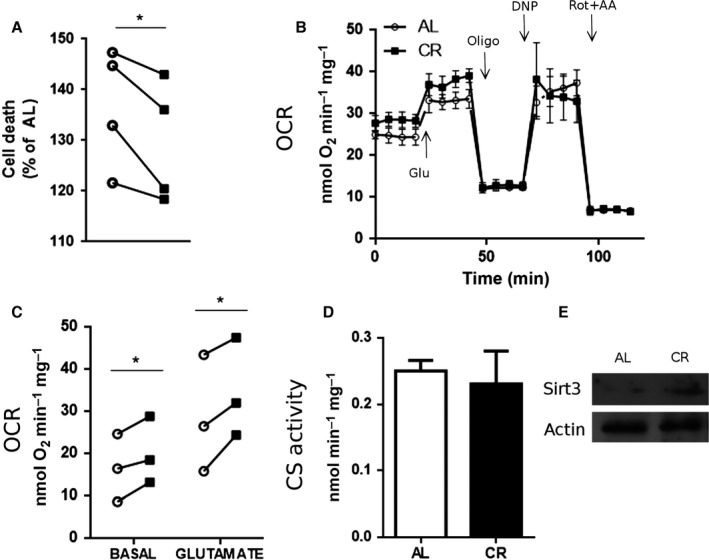
Serum from CR animals protects against excitotoxicity and increases mitochondrial respiration *in vitro*. Cerebellar granule neurons were treated for 24 h with either AL (white bars/symbols) or CR serum (black bars/symbols) and their bioenergetic properties studied. Metabolic parameters were determined as indicated in the Experimental procedures section. (A) Cell death after glutamate challenge. (B) Typical experiment measuring oxygen consumption rates (OCR) using the Seahorse XF Analyzer. Drugs were injected at indicated time points. (C) OCR quantification under basal conditions and after glutamate stimulation. (D) Citrate synthase activity. (E) Western blot for Sirt3 using actin as loading control. Results are shown as mean ± SEM. *P < 0.05 two‐tailed, paired Student's *t*‐test.

Treating cultured cells with serum from CR animals has been shown to reproduce many of the effects observed in the animals, providing an excellent model to study CR *in vitro* (de Cabo *et al*., 2003; Cerqueira *et al*., 2012). Indeed, when primary cerebellar granule neurons (CGNs) were incubated for 24 h with serum from CR rats and then acutely challenged with glutamate to promote excitotoxicity, they showed increased protection against the insult relative to cells incubated in AL serum (Fig. 4A). This result shows that the protective effects of CR against excitotoxicity occur directly on neurons, and do not require the presence of glia or involve changes in the metabolism of the excitatory stimulant.

Cellular conditions such as these are ideal to analyze the mitochondrial effects of CR on excitotoxicity in a real‐time manner. Figure 4B shows a typical oxygen consumption flux analysis trace from cells treated with each serum. We found that CR serum consistently increased basal and glutamate‐stimulated respiratory rates in CGNs (Fig. 4B,C), but did not alter mitochondrial coupling (as indicated by equal rates in the presence of oligomycin, Fig. 4B). Similarly to the *in vivo* situation, we found no changes in citrate synthase activity (Fig. 4D) and an increase in the levels of Sirt3 in cultured neurons treated with CR serum (Fig. 4E). These results indicate that the metabolic and protective effects induced by CR can be mimicked by CR serum and suggest the existence of circulating metabolites in these animals able to induce these changes. Furthermore, they demonstrate that enhanced mitochondrial activity occurs *in situ*, both under basal and excitotoxic conditions.

### CR promotes CypD deacetylation and inhibition, enhancing calcium retention capacity in brain mitochondria

**Figure 5:**
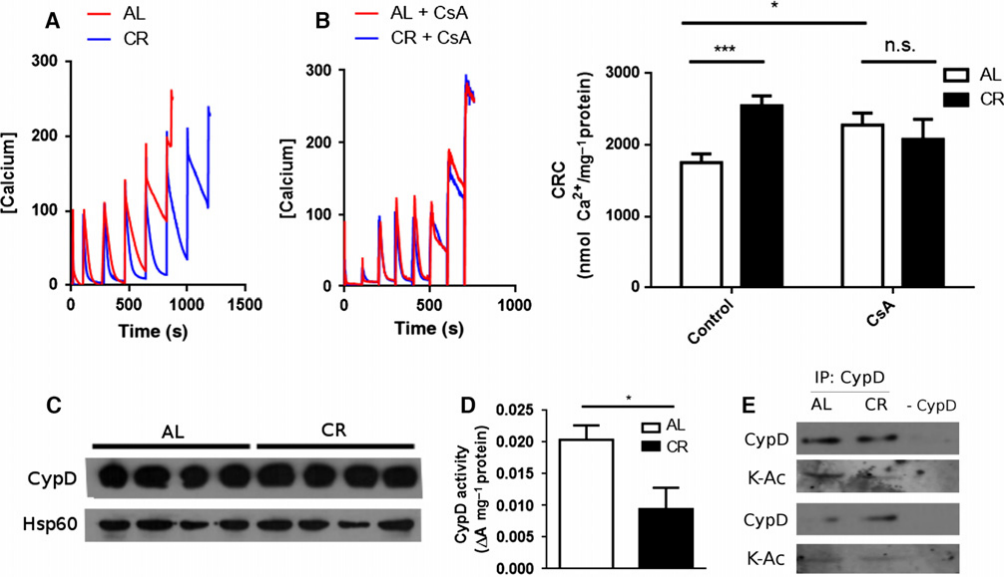
Calcium retention capacity is increased in brains from CR animals. Calcium uptake in brain mitochondria from AL or CR animals was monitored using Calcium Green‐5N as described in Experimental procedures. (A) Representative traces. Each peak corresponds to a 100 μm calcium addition. (B) Representative traces in the presence of 5 μm cyclosporin A (CsA). (C) Quantification of calcium retention capacity (CRC). (D) Western blot against cyclophilin D (CypD) using Hsp60 as loading control. (E) Peptidyl prolyl isomerase activity from brain AL or CR mitochondrial fractions. (F) Immunoprecipitation of mitochondrial samples from AL and CR brains with anti‐CypD antibody and blotting using anti‐CypD and anti‐acetylated lysine antibodies. The negative control (‐ CypD) was performed without anti‐CypD. Results are shown as mean ± SEM. *P < 0.05; ***P < 0.001 two‐tailed, unpaired Student's *t*‐test.

Increased mitochondrial activity during excitotoxicity may be related to changes in calcium buffering by these organelles, which can accumulate large quantities of calcium in a membrane potential‐driven manner, thereby decreasing deleterious levels of this ion in the cytoplasm. The amount of calcium that can be accumulated is limited by the occurrence of mitochondrial permeability transition (MPT), an event in which the inner mitochondrial membrane becomes permeant to solutes under 1500 Da, causing the disruption of the proton gradient and oxidative phosphorylation, in addition to the release of pro‐apoptotic factors (Halestrap, 2009). Increased mitochondrial calcium buffering capacity has been shown to delay MPT and to protect against excitotoxic stimuli (Schinzel *et al*., 2005; Li *et al*., 2009). Based on this knowledge, we evaluated the capacity of isolated brain mitochondria from CR and AL animals to take up and accumulate calcium. Figure 5A shows typical calcium uptake traces in which calcium was added at equal intervals until the accumulation capacity was saturated, and no uptake (seen as a decrease in the measured extramitochondrial calcium levels) could be observed. We found that brain mitochondria from CR rats showed two significant changes in calcium handling: (i) They presented higher capacity to accumulate calcium (calcium retention capacity, CRC), as indicated by the larger number of calcium additions to reach saturation (Fig. 5A, quantified in 5B); (ii) CR mitochondria had faster calcium uptake rates following the first calcium addition. CRC can be increased using cyclosporin A (CsA), which inhibits peptidyl prolyl isomerase cyclophilin D (CypD), the best characterized MPT regulator. Interestingly, CsA did not increase CRC in CR brain mitochondria, but enhanced both CRC and calcium uptake rates in mitochondria from AL animals to levels comparable to those of CR mitochondria (Fig. 5A–C).

Enhanced CRC and insensitivity to CsA are mitochondrial traits observed in CypD‐deficient mice (Basso *et al*., 2005; Nakagawa *et al*., 2005; Schinzel *et al*., 2005). We therefore hypothesized that CR could be decreasing the levels of CypD. However, we did not observe any changes in the protein levels of CypD in CR brain mitochondria relative to AL (Fig. 5D). Interestingly, despite the lack of a change in CypD levels, the peptidyl prolyl isomerase activity of CypD was lower in mitochondria from CR brains (Fig. 5E). CypD is a target of many posttranslational modifications which affect its activity, including deacetylation by Sirt3, a modification which is associated with decreased CypD activity (Wei *et al*., 2013; Bochaton *et al*., 2015). As we observe an increase in Sirt3 in CR, we evaluated acetylation levels by immunoprecipitating CypD from CR and AL brain mitochondria and blotting with an antibody against acetylated lysines. CypD from CR mitochondria showed lower acetylation levels compared with AL (Fig. 5F). Our results therefore indicate that CR increases CRC in brain mitochondria by decreasing the activity of CypD through deacetylation, possibly mediated by Sirt3.

## Discussion

Excessive glutamate spilling due to neuronal death or deficient neurotransmitter clearance produced by energy deficits results in overactivation of postsynaptic receptors and excitotoxicity, a process that participates in neurodegeneration in many diseases. In excitatory neurons, the activation of NMDA receptors produces an increase in intracellular sodium and calcium that triggers a cascade of events resulting in neuronal death. Mitochondria play a pivotal role in excitotoxicity, acting as high‐capacity calcium buffers; cell death occurs when this buffering capacity is overwhelmed (Nicholls, 2009).

CR has been shown to improve lifespan and health span and to protect against many brain pathologies, although the molecular mechanisms involved are far from clear. Here we show that CR is also effective in preventing direct excitotoxic damage (Figs 1 and 4). Our data show that mitochondria in the brains of CR animals have enhanced electron transport capacity, accompanied by higher levels of some electron transport proteins and proteins involved in mitochondrial morphology and dynamics (Fig. 3). Interestingly, the increase in ETC. enzyme activities does not seem to affect the respiratory rates of isolated mitochondria. It is well established that the control of ATP turnover and substrate oxidation in isolated mitochondria is widely distributed among the different components of oxidative phosphorylation and that the regulation exerted by each component is dependent on the conditions chosen (Brand & Nicholls, 2011). Thus, we cannot rule out that (i) respiration is increased *in vivo* and/or (ii) that the higher ETC. enzyme activities observed in CR brain mitochondria only become evident in specific circumstances, such as bioenergetic dysfunction.

Importantly, the results presented here and by others suggest that the term ‘mitochondrial mass’ should be used with caution. Cells seem to be able to regulate independently many different mitochondrial features. In our case, CR increases the levels of cardiolipin in the brain, while the activity of citrate synthase remains constant. Moreover, some, but not all, mitochondrial proteins are enriched in a per mitochondrion basis after CR (Fig. 3).

Some of the metabolic adaptations that CR induces in the brain seem to be mediated by molecule(s) present in the bloodstream. Indeed, CR serum promotes mitochondrial adaptations in primary neurons analogous those observed *in vivo,* namely protection against glutamate excitotoxicity (Fig. 4). Previous reports in other tissues indicate that metabolic effects observed with CR can be partly reproduced *in vitro* using serum from animals subjected to the diet (de Cabo *et al*., 2003). These results support the notion that the metabolic remodeling that takes place with CR can be triggered by circulating molecules. A possible candidate is adiponectin, which is elevated in CR animals (Cerqueira *et al*., 2012). Adiponectin protects against excitotoxicity both *in vivo* (Jeon *et al*., 2009) and *in vitro* (Qiu *et al*., 2011), and removing it from the serum abrogates the activation of the oxidative phosphorylation‐regulating eNOS pathway promoted by CR in vascular cells (Cerqueira *et al*., 2012).

Oxidative phosphorylation directly affects mitochondrial calcium transport properties because the uptake of this ion is energetically driven by the electrochemical gradient. Despite this, and the knowledge that mitochondrial calcium transport is centrally involved in excitotoxicity and many age‐related diseases, the effects of CR on brain mitochondrial calcium uptake have not been explored to date. Hofer *et al*. (2009) demonstrated that susceptibility to MPT in heart mitochondria from aged rats maintained under CR was lower. Our results in brain mitochondria show similarly that CR promotes sizable increases in both the rate and the accumulation capacity for calcium (Fig. 5). As a result, under excitotoxic conditions, CR neurons possess a largely enhanced ability to buffer cytosolic calcium levels, which explains the strong resistance toward excitotoxic damage conferred by this dietary intervention both *in vitro* and *in vivo* (Figs 1 and 4).

The difference in CRC and uptake rates between CR and AL brain mitochondria is abrogated in the presence of cyclosporin A (Fig. 5), indicating a role for CypD in the adaptation of brain mitochondria to CR. Indeed, CypD activity was strongly decreased by CR, while, interestingly, protein levels were unchanged (Fig. 5). This suggests that a posttranslational modification of CypD occurs in a manner regulated by dietary interventions. Because CR increased the levels of the mitochondrial deacetylase Sirt3 (Figs 3 and 4), and CypD is activated by acetylation (Wei *et al*., 2013; Bochaton *et al*., 2015), we hypothesized that CypD may be less active in CR due to deacetylation, as confirmed by immunoprecipitation followed by blotting for acetylation (Fig. 5F). Our results are in line with studies showing that the overexpression of mutated forms of CypD that mimic constitutive acetylated or de‐acetylated states, respectively, decrease and increase CRC in MEFs (Bochaton *et al*., 2015). Also, expression of Sirt3 in cultured mouse cortical neurons is increased in response to excitotoxic NMDA and exerts a protective role (Kim *et al*., 2011). Moreover, Sirt3 knockout mice are more susceptible to excitotoxicity; their brain mitochondria are more prone to MPT and CypD acetylation levels are higher (Cheng *et al*., 2015). Finally, CR‐induced expression of Sirt3 had been previously described in other tissues, including white and brown adipose tissues (Shi *et al*., 2005) and skeletal muscle (Palacios *et al*., 2009). Interestingly, although acetylation and deacetylation have been implicated in many regulatory processes in mitochondria (Cimen *et al*., 2010; Kong *et al*., 2010; Qiu *et al*., 2010; Ozden *et al*., 2014; Tao *et al*., 2014; Liu *et al*., 2015; Traba *et al*., 2015), a recent report (Weinert *et al*., 2015) has challenged the importance of this modification as a significant regulatory mechanism, because protein acetylation is a rare event, with a median occupancy of acetylation sites on mitochondrial proteins as low as 0.11%. These authors also measured acetylation in response to fasting and found no significant changes in whole tissue acetylation in brain. However, as pointed out in a comment on the paper by Lombard *et al*. (2015), low‐level acetylation can still have significant important biological consequences when associated with activation of specific proteins, as is the case for CypD. Thus, we propose a mechanism to explain our data in concert with previous observations where CR induces the expression of Sirt3 in mitochondria, which in turn promotes oxidative phosphorylation and inhibits CypD through deacetylation, thereby increasing CRC and protecting against calcium overload in situations such as excitotoxicity.

Overall, we demonstrate that CR is a highly effective intervention to prevent excitotoxic neuronal cell death by enhancing antioxidant capacity, mitochondrial respiratory rates, preventing MPT and thus enhancing calcium accumulation capacity, resulting in lower cell death. These properties may be central to the mechanism through which this dietary intervention promotes its many beneficial neurological effects.

## Experimental procedures

### Animals, caloric restriction, and serum collection

All experiments were conducted in agreement with the National Institutes of Health guidelines for humane treatment of animals and were reviewed and approved by the local animal care and use committee (protocol 17/2013). Adult Swiss mice (2 months of age) and Sprague Dawley rats (2 months of age) were fed either *ad libitum* (free access to AIN‐93‐M diet prepared by Rhoster, Campinas, SP, Brazil) or 40% CR using a diet supplemented with vitamins and micronutrients to avoid malnutrition. CR feedings were adjusted weekly by weight based on AL food consumption measured 1 week earlier. Food was offered to CR mice every day at 10:00 am. The animals were lodged five individuals per cage and given water *ad libitum*. The night before sacrificing the animals, food was removed from the control group, which was otherwise fed *ad libitum*. To collect serum, animals were anesthetized using a mixture of ketamine and xylazine and the blood was collected from the cava vein in heparin‐treated tubes and centrifuged. Sera from animals within the same treatment regimen were pooled, aliquoted, and kept at −20 °C until use.

### Kainic acid treatment

Two‐month‐old Swiss male mice were kept on a 40% CR diet for 14 weeks. Forty‐eight hours before the experiment, animals were housed in individual cages, and food was removed after 24 h. Kainate administration was performed intraperitoneally using a concentration of 25 mg kg^−1^ and seizure appearance was followed for 2 h using Racine's table.

### Cardiolipin quantification

To extract the phospholipids from whole‐brain homogenates, 500 μL of sample was incubated with 1 mL 1 m KCl, 1 mL methanol, and 2 mL chloroform and incubated for 30 min under vigorous shaking. Afterward, 1 mL of chloroform and 0.5 mL of methanol were added and the emulsion was centrifuged at 800 g for 20 min. The lower organic phase was separated and nitrogen‐dried. Lipids were dissolved in 100 μL chloroform:isopropanol (1:1). Cardiolipin quantification was performed by thin‐layer chromatography. Total lipid extracts (100 μg) were applied on a Kieselguhr silica matrix pretreated with 1.8% boric acid in ethanol. Plates were baked for 1 h at 100 °C and developed in chloroform:methanol:NH_4_OH (28% solution):H_2_O (60:37.5:3:1). Spots were visualized by iodine staining and analyzed by densitometry using bovine heart cardiolipin as standard.

### Mitochondrial isolation

Brain mitochondria from overnight fasted rats were isolated by differential centrifugation as previously described (Tahara *et al*., 2009). The final pellet was resuspended in experimental medium (75 mm d‐mannitol, 25 mm sucrose, 5 mm KH_2_PO_3_, 20 mm Tris–HCl, 0.5 mm EDTA, 100 mm KCl, and 0.1% BSA free of fatty acids) supplemented with 1 mm EGTA.

### Enzymatic activities

Glutathione peroxidase and glutathione reductase activities were measured as described by Mantha *et al*. (1993). Superoxide dismutase activity was measured in mitochondrial fractions as previously described (Weydert & Cullen, 2010). Electron transport chain activities were measured as described in Spinazzi *et al*. (2012).

### Oxygen consumption in isolated mitochondria

Oxygen consumption was measured in experimental medium supplemented with 1 mm EGTA using an Oroboros system. Pyruvate plus malate (5 mm each) were used as substrates for complex I, whereas rotenone (2 μm) plus succinate (5 mm) for complex II. State 4 was determined after the addition of 0.5 μg mL^−1^ oligomycin and uncoupled respiration determined after titration of CCCP until maximal respiration was achieved (1–2 μm).

### Western blotting and antibodies

Mitochondrial fractions were resolved by SDS–PAGE using 10 or 12% gels and transferred to nitrocellulose membranes. Detection of specific proteins was achieved using the following antibodies and dilutions: NDUFS3 (1:5000), UQCRC2 (1:5000), and ATPB (1:5000; Mitosciences, Eugene, OR, USA); Mfn‐2 (1:5000), Drp‐1 (1:5000), acetylated lysine (1:1000), and Sirt3 (1:2000; Cell Signaling, Denvers, MA, USA); cyclophilin D (1:5000) and Hsp60 (1:5000; Abcam, Cambridge, UK).

### Calcium retention capacity and calcium uptake rates

Mitochondria (200–400 μg) were suspended in a cuvette containing 2 mL of experiment buffer supplemented with 1 mm succinate, 2 μm rotenone, 1 μm MgCl_2_, 1 mm ATP, and 0.1 μm Calcium Green 5N. Fluorescence was monitored using a F‐2500 Hitachi (Chiyoda, Tokyo, Japan) Fluorimeter at 30 °C with a 506‐nm excitation and 532‐nm emission wavelengths. Calcium additions (100 μm) were made every 100 s. Uptake rates were determined as the slope of the first calcium addition. Saturating concentrations of calcium and EGTA were subsequently added at the end of each experiment to obtain maximum fluorescence and minimum fluorescence, respectively. Calcium concentrations were calculated at each time point using the formula [Ca^2+^] = *K*_d_ × (*F* – *F*_min_)/(*F*_max_ – *F*). The *K*_d_ was empirically determined using the first addition of 100 μm.

### Cyclophilin D activity

Prolyl peptidyl isomerase activity of cyclophilin D was determined colorimetrically in mitochondrial fractions (10–15 μg per assay) using the synthetic peptide N‐succinyl‐Ala‐Ala‐Pro‐Phe‐p‐nitroanilide as previously described (Fortes *et al*., 2001). Conversion of the Ala‐Pro bond from *cis* to *trans* by cyclophilin renders the peptide susceptible to enzymatic cleavage by chymotrypsin, releasing the chromogenic dye p‐nitroanilide. The reaction was carried out in Hepes‐K^+^ buffer pH 8.0 and monitored measuring the absorbance change at 390 nm for 2 min. Background activity (i.e. formation of p‐nitroanilide in the absence of mitochondria) was subtracted from all samples.

### Immunoprecipitation

Mitochondrial homogenates (0.75 mg) were incubated with or without anti‐cyclophilin D antibody (5 μg) at 4 °C for 3 h under gentle agitation; 50 μL of protein A‐agarose (Sigma‐Aldrich, St. Louis, MO, USA) were added to each sample and incubated overnight under the same conditions. The next day, samples were washed 5–6 times in IP buffer (phosphate buffer pH 8.0, 0.5% Triton X‐100, 0.1 mm NaCl), treated with 30 μL of Laemli buffer, and heated at 100 °C for 5 min. The resulting supernatants were then resolved in a 12% SDS–PAGE and incubated with antibodies raised against anti‐cyclophilin D (Mitosciences, 1:5000, made in mouse) and anti‐acetyl‐lysine (Cell Signaling, 1:1000, made in rabbit). To circumvent the problems associated with antibody stripping and to prevent false positives, we developed the cyclophilin D signal using a mouse HRP‐conjugated secondary antibody followed by ECL detection, and the acetylated lysine signal using a rabbit 680 infrared antibody and an Odyssey Imaging system.

### Cerebellar granule cell cultures and glutamate treatment

Primary cerebellar granule neurons were obtained from P6 Sprague Dawley rats. Cerebella from five male pups were removed and pooled together in 18 mL of dissection medium (PBS, 1% BSA, 5 mm glucose) and treated with 0.006% trypsin for 10 min at 37 °C. Trypsin was inactivated by adding 2 mL of fetal bovine serum to a final concentration of 10%. Cerebella were centrifuged for 5 min at 200 g, and the supernatant was discarded. The pellet was resuspended in 3–4 mL of fresh dissection medium and mechanically minced using fire‐polished Pasteur pipettes of different widths. The medium was completed to 10 mL, filtered through a 70‐μm‐diameter nylon filter and centrifuged as before. The resulting pellet was resuspended in Neurobasal medium with B27, glutamax, and 25 mm KCl and seeded in poly‐lysine‐coated plates to a final density of 100 000 cells per well. Partial medium changes were performed at DIV3 (day *in vitro* 3) and DIV5. On DIV7, the medium was replaced with media supplemented with *ad libitum* (AL) or CR serum to a final volume of 300 μL and a final serum concentration of 10%. Acute glutamate treatment was performed at DIV8, replacing the culture medium with Locke's solution (134 mm NaCl, 25 mm KCl, 4 mm NaHCO_3_, 5 mm Hepes, 2.3 mm CaCl_2_, 5 mm glucose) for 30 min at 37 °C, after which cells were put back in their conditioned media with a 20% added volume of fresh medium. Cell death was assayed 24 h later using lactate dehydrogenase assay as described below.

### Oxygen consumption measurements in attached cells

Cerebellar granule cells were obtained as described above and seeded in Seahorse XF24 V7 plates. On DIV8 (day *in vitro*), the medium was partially replaced with fresh medium with serum from either AL or CR animals to a final concentration of 10%. Oxygen consumption rates (OCR) were determined using a Seahorse XF24 equipment on DIV9. One hour prior to the experiment, the medium was replaced with DMEM without bicarbonate and pyruvate and supplemented with KCl to a final concentration of 25 mm. The first injector was loaded with 100 μm glutamate, the second with 1 μg mL^−1^ oligomycin, the third with 0.5 mm 2‐dinitrophenol (DNP), and the fourth with 1 μm rotenone plus 1 μm antimycin A. At the end of each experiment, cells were washed twice with PBS and protein content was determined in each well to normalize OCR values. Nonmitochondrial respiration (i.e. the OCR after the addition of rotenone plus antimycin) was subtracted from all measurements. The proton leak was calculated as the percentage of basal respiration that was not inhibited by oligomycin.

### Cell death measurements

Cell death was measured by determining lactate dehydrogenase (LDH) activity in the medium. Aliquots (50 μL) of cell media were assayed using 100 μL of a reaction mixture containing 4.2 mm sodium pyruvate, 0.75 mm NADH, and 9.45 mm NaHCO_3_. The reaction was monitored using a plate reader following the decrease in absorbance at 340 nm. Data from each well was normalized by maximal activity after permeabilization with 0.2% Triton X‐100.

### Data analysis and statistics

Results were compared using two‐tailed Student's *t*‐tests, either paired or unpaired as indicated in the figure legends.

## Author contributions

I.A. and A.K. designed research; I.A., S.M.‐F., L.A.L.‐M., and B.C. performed research and analyzed data; I.A. and A.K. wrote the manuscript.

## Funding

This work was supported by grants from the Fundação de Amparo à Pesquisa do Estado de São Paulo (FAPESP, 2010/51906‐1) Centro de Pesquisa, Inovacão e Difusão de Processos Redox em Biomedicina (13/07937‐8), Núcleo de Pesquisa em Processos Redox em Biomedicina (NAP‐Redoxoma), Instituto Nacional de Ciência e Tecnologia de Processos Redox em Biomedicina (INCT Redoxoma), and Conselho Nacional de Pesquisa e Desenvolvimento (CNPq, 153560/2011‐8, 302898/2013‐1). I. A. is a recipient of a FAPESP Postdoctoral Fellowship (2012/51288‐1).

## Conflict of interest

None declared.

## References

[acel12527-bib-0001] Amigo I , Kowaltowski AJ (2014) Dietary restriction in cerebral bioenergetics and redox state. Redox. Biol. 2, 293–304. 10.1016/j.redox.2013.12.021 PMC3926116 24563846

[acel12527-bib-0002] Basso E , Fante L , Fowlkes J , Petronilli V , Forte MA , Bernardi P (2005) Properties of the permeability transition pore in mitochondria devoid of cyclophilin D. J. Biol. Chem. 280, 18558–18561. 15792954 10.1074/jbc.C500089200

[acel12527-bib-0003] Bochaton T , Crola‐Da‐Silva C , Pillot B , Villedieu C , Ferreras L , Alam MR , Thibault H , Strina M , Gharib A , Ovize M , Baetz D (2015) Inhibition of myocardial reperfusion injury by ischemic postconditioning requires sirtuin 3‐mediated deacetylation of cyclophilin D. J. Mol. Cell. Cardiol. 84, 61–69. 25871830 10.1016/j.yjmcc.2015.03.017

[acel12527-bib-0004] Brand MD , Nicholls DG (2011) Assessing mitochondrial dysfunction in cells. Biochem J. 435, 297–312. 21726199 10.1042/BJ20110162 PMC3076726

[acel12527-bib-0005] Bruce‐Keller AJ , Umberger G , McFall R , Mattson MP (1999) Food restriction reduces brain damage and improves behavioral outcome following excitotoxic and metabolic insults. Ann. Neurol. 45, 8–15. 9894871

[acel12527-bib-0006] de Cabo R , Fürer‐Galbán S , Anson RM , Gilman C , Gorospe M , Lane MA (2003) An *in vitro* model of caloric restriction. Exp. Gerontol. 38, 631–639. 12814798 10.1016/s0531-5565(03)00055-x

[acel12527-bib-0007] Cerqueira FM , Brandizzi LI , Cunha FM , Laurindo FRM , Kowaltowski AJ (2012) Serum from calorie‐restricted rats activates vascular cell eNOS through enhanced insulin signaling mediated by adiponectin. PLoS ONE 7, e31155. 22319612 10.1371/journal.pone.0031155 PMC3271099

[acel12527-bib-0008] Cheng A , Yang Y , Zhou Y , Maharana C , Lu D , Peng W , Liu Y , Wan R , Marosi K , Misiak M , Bohr VA , Mattson MP (2015) Mitochondrial SIRT3 mediates adaptive responses of neurons to exercise and metabolic and excitatory challenges. Cell Metab. 23, 128–142. 26698917 10.1016/j.cmet.2015.10.013 PMC5141613

[acel12527-bib-0009] Cimen H , Han M‐J , Yang Y , Tong Q , Koc H , Koc EC (2010) Regulation of succinate dehydrogenase activity by SIRT3 in mammalian mitochondria. Biochemistry 49, 304–311. 20000467 10.1021/bi901627u PMC2826167

[acel12527-bib-0010] Contestabile A , Ciani E , Contestabile A (2004) Dietary restriction differentially protects from neurodegeneration in animal models of excitotoxicity. Brain Res. 1002, 162–166. 14988047 10.1016/j.brainres.2004.01.005

[acel12527-bib-0011] Fontana L , Partridge L (2015) Promoting health and longevity through diet: from model organisms to humans. Cell 161, 106–118. 25815989 10.1016/j.cell.2015.02.020 PMC4547605

[acel12527-bib-0012] Fortes F , Castilho RF , Catisti R , Carnieri EG , Vercesi AE (2001) Ca^2+^ induces a cyclosporin A‐insensitive permeability transition pore in isolated potato tuber mitochondria mediated by reactive oxygen species. J. Bioenerg. Biomembr. 33, 43–51. 11460925 10.1023/a:1005672623709

[acel12527-bib-0013] Froy O , Miskin R (2010) Effect of feeding regimens on circadian rhythms: implications for aging and longevity. Aging 2, 7–27. 20228939 10.18632/aging.100116 PMC2837202

[acel12527-bib-0014] Halestrap AP (2009) What is the mitochondrial permeability transition pore? J. Mol. Cell. Cardiol. 46, 821–831. 19265700 10.1016/j.yjmcc.2009.02.021

[acel12527-bib-0015] Hancock CR , Han D‐H , Higashida K , Kim SH , Holloszy JO (2011) Does calorie restriction induce mitochondrial biogenesis? A reevaluation FASEB J. 25, 785–791. 21048043 10.1096/fj.10-170415 PMC3023396

[acel12527-bib-0016] Hofer T , Servais S , Seo AY , Marzetti E , Hiona A , Upadhyay SJ , Wohlgemuth SE , Leeuwenburgh C (2009) Bioenergetics and permeability transition pore opening in heart subsarcolemmal and interfibrillar mitochondria: effects of aging and lifelong calorie restriction. Mech. Ageing Dev. 130, 297–307. 19428447 10.1016/j.mad.2009.01.004 PMC2680750

[acel12527-bib-0017] Jeon BT , Shin HJ , Kim JB , Kim YK , Lee DH , Kim KH , Kim HJ , Kang SS , Cho GJ , Choi WS , Roh GS (2009) Adiponectin protects hippocampal neurons against kainic acid‐induced excitotoxicity. Brain Res. Rev. 61, 81–88. 19460404 10.1016/j.brainresrev.2009.05.002

[acel12527-bib-0018] Kim SH , Lu HF , Alano CC (2011) Neuronal Sirt3 protects against excitotoxic injury in mouse cortical neuron culture. PLoS ONE 6, e14731. 21390294 10.1371/journal.pone.0014731 PMC3046953

[acel12527-bib-0019] Kong X , Wang R , Xue Y , Liu X , Zhang H , Chen Y , Fang F , Chang Y (2010) Sirtuin 3, a new target of PGC‐1alpha, plays an important role in the suppression of ROS and mitochondrial biogenesis. PLoS ONE 5, e11707. 20661474 10.1371/journal.pone.0011707 PMC2908542

[acel12527-bib-0020] Lanza IR , Zabielski P , Klaus KA , Morse DM , Heppelmann CJ , Bergen HR , Dasari S , Walrand S , Short KR , Johnson ML , Robinson NM , Schimke JM , Jakaitis DR , Asmann YW , Sun Z , Nair KS (2012) Chronic caloric restriction preserves mitochondrial function in senescence without increasing mitochondrial biogenesis. Cell Metab. 16, 777–788. 23217257 10.1016/j.cmet.2012.11.003 PMC3544078

[acel12527-bib-0021] Li V , Brustovetsky T , Brustovetsky N (2009) Role of cyclophilin D‐dependent mitochondrial permeability transition in glutamate‐induced calcium deregulation and excitotoxic neuronal death. Exp. Neurol. 218, 171–182. 19236863 10.1016/j.expneurol.2009.02.007 PMC2710407

[acel12527-bib-0022] Lin A‐L , Coman D , Jiang L , Rothman DL , Hyder F (2014) Caloric restriction impedes age‐related decline of mitochondrial function and neuronal activity. J. Cereb. Blood Flow Metab. 34, 1440–1443. 24984898 10.1038/jcbfm.2014.114 PMC4158670

[acel12527-bib-0023] Liu L , Peritore C , Ginsberg J , Kayhan M , Donmez G (2015) SIRT3 attenuates MPTP‐induced nigrostriatal degeneration via enhancing mitochondrial antioxidant capacity. Neurochem. Res. 40, 600–608. 25555707 10.1007/s11064-014-1507-8

[acel12527-bib-0024] Lombard DB , Alt FW , Cheng H‐L , Bunkenborg J , Streeper RS , Mostoslavsky R , Kim J , Yancopoulos G , Valenzuela D , Murphy A , Yang Y , Chen Y , Hirschey MD , Bronson RT , Haigis M , Guarente LP , Farese RV Jr , Weissman S , Verdin E , Schwer B (2007) Mammalian Sir2 homolog SIRT3 regulates global mitochondrial acetylation. Mol. Cell. Biol. 27, 8807–8814. 17923681 10.1128/MCB.01636-07 PMC2169418

[acel12527-bib-0025] Lombard DB , Dash BP , Kumar S (2015) Acetyl‐ed question in mitochondrial biology? EMBO J. 34, 2597–2600. 26369717 10.15252/embj.201592927 PMC4641526

[acel12527-bib-0026] Mantha SV , Prasad M , Kalra J , Prasad K (1993) Antioxidant enzymes in hypercholesterolemia and effects of vitamin E in rabbits. Atherosclerosis 101, 135–144. 8379958 10.1016/0021-9150(93)90110-g

[acel12527-bib-0027] Nakagawa T , Shimizu S , Watanabe T , Yamaguchi O , Otsu K , Yamagata H , Inohara H , Kubo T , Tsujimoto Y (2005) Cyclophilin D‐dependent mitochondrial permeability transition regulates some necrotic but not apoptotic cell death. Nature 434, 652–658. 15800626 10.1038/nature03317

[acel12527-bib-0028] Nicholls DG (2009) Mitochondrial calcium function and dysfunction in the central nervous system. Biochim. Biophys. Acta 1787, 1416–1424. 19298790 10.1016/j.bbabio.2009.03.010 PMC2752662

[acel12527-bib-0029] Ozden O , Park S‐H , Wagner BA , Yong Song H , Zhu Y , Vassilopoulos A , Jung B , Buettner GR , Gius D (2014) SIRT3 deacetylates and increases pyruvate dehydrogenase activity in cancer cells. Free Radic. Biol. Med. 76, 163–172. 25152236 10.1016/j.freeradbiomed.2014.08.001 PMC4364304

[acel12527-bib-0030] Palacios OM , Carmona JJ , Michan S , Chen KY , Manabe Y , Ward JL , Goodyear LJ , Tong Q (2009) Diet and exercise signals regulate SIRT3 and activate AMPK and PGC‐1alpha in skeletal muscle. Aging 1, 771–783. 20157566 10.18632/aging.100075 PMC2815736

[acel12527-bib-0031] Qiu X , Brown K , Hirschey MD , Verdin E , Chen D (2010) Calorie restriction reduces oxidative stress by SIRT3‐mediated SOD2 activation. Cell Metab. 12, 662–667. 21109198 10.1016/j.cmet.2010.11.015

[acel12527-bib-0032] Qiu G , Wan R , Hu J , Mattson MP , Spangler E , Liu S , Yau S‐Y , Lee TMC , Gleichmann M , Ingram DK , So KF , Zou S (2011) Adiponectin protects rat hippocampal neurons against excitotoxicity. Age 33, 155–165. 20842535 10.1007/s11357-010-9173-5 PMC3127462

[acel12527-bib-0033] Schinzel AC , Takeuchi O , Huang Z , Fisher JK , Zhou Z , Rubens J , Hetz C , Danial NN , Moskowitz MA , Korsmeyer SJ (2005) Cyclophilin D is a component of mitochondrial permeability transition and mediates neuronal cell death after focal cerebral ischemia. Proc. Natl Acad. Sci. USA 102, 12005–12010. 16103352 10.1073/pnas.0505294102 PMC1189333

[acel12527-bib-0034] Sharma S , Kaur G (2005) Neuroprotective potential of dietary restriction against kainate‐induced excitotoxicity in adult male Wistar rats. Brain Res. Bull. 67, 482–491. 16216697 10.1016/j.brainresbull.2005.07.015

[acel12527-bib-0035] Shi T , Wang F , Stieren E , Tong Q (2005) SIRT3, a mitochondrial sirtuin deacetylase, regulates mitochondrial function and thermogenesis in brown adipocytes. J. Biol. Chem. 280, 13560–13567. 15653680 10.1074/jbc.M414670200

[acel12527-bib-0036] Spinazzi M , Casarin A , Pertegato V , Salviati L , Angelini C (2012) Assessment of mitochondrial respiratory chain enzymatic activities on tissues and cultured cells. Nat. Protoc. 7, 1235–1246. 22653162 10.1038/nprot.2012.058

[acel12527-bib-0037] Stewart J , Mitchell J , Kalant N (1989) The effects of life‐long food restriction on spatial memory in young and aged Fischer 344 rats measured in the eight‐arm radial and the Morris water mazes. Neurobiol. Aging 10, 669–675. 2628778 10.1016/0197-4580(89)90003-1

[acel12527-bib-0038] Tahara EB , Navarete FDT , Kowaltowski AJ (2009) Tissue‐, substrate‐, and site‐specific characteristics of mitochondrial reactive oxygen species generation. Free Radic. Biol. Med. 46, 1283–1297. 19245829 10.1016/j.freeradbiomed.2009.02.008

[acel12527-bib-0039] Tao R , Vassilopoulos A , Parisiadou L , Yan Y , Gius D (2014) Regulation of MnSOD enzymatic activity by Sirt3 connects the mitochondrial acetylome signaling networks to aging and carcinogenesis. Antioxid. Redox Signal. 20, 1646–1654. 23886445 10.1089/ars.2013.5482 PMC3942696

[acel12527-bib-0040] Traba J , Kwarteng‐Siaw M , Okoli TC , Li J , Huffstutler RD , Bray A , Waclawiw MA , Han K , Pelletier M , Sauve AA , Siegel RM , Sack MN (2015) Fasting and refeeding differentially regulate NLRP3 inflammasome activation in human subjects. J. Clin. Invest. 125, 4592–4600. 26529255 10.1172/JCI83260 PMC4665779

[acel12527-bib-0041] Wei L , Zhou Y , Dai Q , Qiao C , Zhao L , Hui H , Lu N , Guo Q‐L (2013) Oroxylin A induces dissociation of hexokinase II from the mitochondria and inhibits glycolysis by SIRT3‐mediated deacetylation of cyclophilin D in breast carcinoma. Cell Death Dis. 4, e601. 23598413 10.1038/cddis.2013.131 PMC3641353

[acel12527-bib-0042] Weinert BT , Moustafa T , Iesmantavicius V , Zechner R , Choudhary C (2015) Analysis of acetylation stoichiometry suggests that SIRT3 repairs nonenzymatic acetylation lesions. EMBO J. 34, 2620–2632. 26358839 10.15252/embj.201591271 PMC4641529

[acel12527-bib-0043] Weydert CJ , Cullen JJ (2010) Measurement of superoxide dismutase, catalase and glutathione peroxidase in cultured cells and tissue. Nat. Protoc. 5, 51–66. 20057381 10.1038/nprot.2009.197 PMC2830880

[acel12527-bib-0044] Witte AV , Fobker M , Gellner R , Knecht S , Flöel A (2009) Caloric restriction improves memory in elderly humans. Proc. Natl Acad. Sci. USA 106, 1255–1260. 19171901 10.1073/pnas.0808587106 PMC2633586

